# d‐Amphetamine and Feeding States Cohesively Affect Locomotion and Motor Neuron Response in Zebrafish Larvae

**DOI:** 10.1002/brb3.70173

**Published:** 2024-12-06

**Authors:** Pushkar Bansal, Mitchell F. Roitman, Erica E. Jung

**Affiliations:** ^1^ Department of Mechanical and Industrial Engineering The University of Illinois at Chicago Chicago Illinois USA; ^2^ Department of Psychology The University of Illinois at Chicago Chicago Illinois USA; ^3^ Department of Bioengineering The University of Illinois at Chicago Chicago Illinois USA

**Keywords:** amphetamine, food deprivation, food‐satiation, locomotion, motor neuron activity, zebrafish larvae

## Abstract

**Purpose:**

Amphetamine (AMPH) increases locomotor activities in animals, and the locomotor response to AMPH is further modulated by caloric deficits such as food deprivation and restriction. The increment in locomotor activity regulated by AMPH‐caloric deficit concomitance can be further modulated by varying feeding schedules (e.g., acute and chronic food deprivation and acute feeding after chronic food deprivation). However, the effects of different feeding schedules on AMPH‐induced locomotor activity are yet to be explicated. Here, we have explored the stimulatory responses of acutely administered D‐amphetamine in locomotion under systematically varying feeding states (fed/sated and food deprivation) and schedules (chronic and acute) in zebrafish larvae.

**Method:**

We exposed wild‐type and transgenic [Tg(mnx1:GCaMP5)] zebrafish larvae to 0.7 µM concentration of AMPH and measured swimming activity and spinal motor neuron activity in vivo in real time. The analysis involved time‐elapsed and cumulative manner pre‐ and post‐AMPH treatment in four different caloric states including acute and chronic schedules of feeding and hunger. Both locomotor and motor neuron activities were compared in all four states in both fish lines.

**Findings:**

Our results show that locomotion and motor neuron activity increased in both chronic and acute food deprivation post‐AMPH treatment cumulatively. A steady increase in locomotion was observed in acute food deprivation compared to an immediate abrupt increase in chronic food‐deprivation state. The ad libitum‐fed larvae exhibited a moderate increase both in locomotion and motor neuron activity. Conversely to all other caloric states, food‐sated (acute feeding after chronic food deprivation) larvae moved moderately less and exhibited a mild decrease in motor neuron activity after AMPH treatment.

**Conclusion:**

These results reveal the importance of cohesive effects of feeding schedule and AMPH treatment by revealing the changes in stimulatory characteristics of AMPH on locomotion and motor neuron activity in acute and chronic feeding states.

## Graphical abstract

This preclinical explores the concomitant effect of different feeding states and amphetamine on behavior and brain using larval zebrafish as an animal model. Here, the zebrafish larvae were kept in satiated and food‐deprived state acutely and chronically followed by an acute amphetamine treatment. The findings show that food deprivation significantly potentiated the stimulatory effects of amphetamine compared to fed states by increasing their locomotion and spinal motor neuron activity.

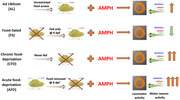

## 1 Introduction

Amphetamine (AMPH), a psychostimulant with a high abuse potential, has been long known to induce abusive compulsivity in humans with its chronic consumption and further leads to addiction and tolerance (Hyman 1996; Berman et al. 2009). A major reason for AMPH abuse is the induction of its reinforcing effects upon its acute and controlled consumption at first, which also promotes physiological activities in humans and animals. Preclinical and clinical studies provide evidence of the modulatory effects of AMPH on the physiological outputs by evoking an increase in motor activities that were observed in animals (Segal and Mandell 1974; Idemudia and McMillan 1984; Yates et al. 2007) and humans (Minassian et al. 2016) whereas intoxication from AMPH abuse has led to severe physiological responses in both animals and humans (Shiorring 1980; Fitzgerald and Bronstein 2013).

The motor response to AMPH can also be affected by the animal's physiological state, such as hunger and satiety. Specifically, food deprivation/restriction is known to enhance the motor response to AMPH. In preclinical studies, Monkeys exhibited an increased AMPH intake in the food‐restricted state, which potentiated AMPH's stimulating effects by increasing their motor activity (Carroll, France, and Meisch 1979). Similarly, the food‐restricted rodents showed enhanced rewarding effects of AMPH by increasing their locomotion (De Vaca and Carr 1998). Other studies involving food‐restricted rodents showed an increased AMPH intake along with higher conditioned place preference (Stuber et al. 2002; Geuzaine et al. 2014). Moreover, acute food restriction for 24 h in rats altered locomotor activity when treated with AMPH (Honma, Kanematsu and Honma 1992; Mabry and Campbell 1975). In these studies, AMPH food restriction‐mediated interactive response was observed when animals were subjected mostly to a chronic food‐restriction state and few to the acute food‐restricted state in separate independent studies. To date, no study has systematically varied the caloric state of the animal by changing the feeding schedule while subjecting them to a caloric‐deficit state, such as acute/chronic food deprivation and satiation acute/ad libitum feeding, to investigate the behavioral and neural motoric effects of AMPH. Research on how food–drug interaction modulates behavior, especially brain activity, remains scarce, and the correlation between behavior and the respective neural substrates in these stimuli is still subject to debate. This research could aid clinicians in developing novel strategies to control the abnormality in behavior and its neural substrates arising from AMPH intake by varying diet of the drug consumers, and time‐elapsed findings would assist strategizing timely interventions for drug abuse circumstances while considering instantaneous feeding state.

Zebrafish larvae are a good animal model for studying motor behavioral and neural responses to addictive drugs. Zebrafish exhibit robust locomotor behavior, and importantly, their optical transparency allows researchers to record and quantify individual motor neuron activity in the spinal cord using genetically encoded neural activity indicators (Muto et al. 2011). Larval zebrafish have shown behavioral sensitivity similar to mammals to a variety of addictive drugs, including AMPH (Irons et al. 2010; Basnet et al. 2019; Cousin et al. 2014; Bansal, Roitman, and Jung 2023). Here, we address whether acute feeding and acute deprivation can differently modulate the motor response to AMPH, especially relative to the ad libitum‐fed and chronically deprived state. In zebrafish larvae, we measured the change in response in behavior and motor neurons in the spinal cord, an underlying neural substrate responsible for motor action, affected by AMPH–caloric stress interaction. To quantify this, we analyzed locomotor and motor neuron activity in a time‐elapsed manner and cumulatively for four different feeding conditions—“ad libitum” (unrestricted food availability), “food sated” (fed 18 h before experimentation), “chronic food deprivation” (never fed) and “acute food deprivation” (18‐h starvation). Our findings showed that chronic and acute food deprivation potentiated the motor‐stimulating effects of AMPHs in zebrafish. Both acute and chronic food deprivation induced a more long‐lasting AMPH‐mediated motor effect where acute food deprivation exhibited progressive increment in activity over intervals, unlike chronic food deprivation. Meanwhile, motor response to AMPH in ad libitum and food‐sated (FS) states was not significant. Overall, our study uncovers how systemic variation in caloric states can alter motor responses by distinctly modulating AMPH's inherent stimulatory response.

## 2 Materials and Methods

Adult wild‐type (abwt) and transgenic Tg(mnx1:GCaMP5) zebrafish were housed at the zebrafish facility. They were maintained in an automated water racking system (Aquaneering Inc., San Diego, CA). All conducted experiments were approved by AALAC, and all guidelines were diligently followed. The Zebrafish facility maintained a 14/10‐h light cycle schedule set at 28°C. All experiments used 7 days post fertilization (dpf) zebrafish larvae. This study involves different feeding schedules to measure larval responses, and zebrafish larvae develop neurons responsible for mediating feeding responses (Wee et al. 2019). Also, in the larval stage, this teleost has been shown to stay healthy without any phenotypical anomaly till 8 dpf (Hernandez et al. 2018). Moreover, the feeding schedule organized for FS and acute food deprivation only allowed us to use 7 dpf larvae for uniformity among all feeding states; thus, 7 dpf seemed appropriate for conducting experiments. Further, the zebrafish rack system was maintained at 28°C with water conductivity within 600–750 µs, and pH was maintained within a range of 7.2–8.0. Larvae in the embryonic stage were initially kept in water with the abovementioned conditions in the fish room in all states (harvesting, hatching, experiments) and were brought to the research facility when they turned 7 dpf.

### 2.1 Experimental Groups

For locomotor activity analysis, we divided zebrafish into two groups, namely, control and AMPH treatment groups. These two groups were further subdivided into eight groups, depending on their feeding states (control: AL‐AMPH [ad libitum fed without AMPH, *n* = 17], FS‐AMPH [food‐sated without AMPH, *n* = 14], CFD‐AMPH [chronic food deprivation without AMPH, *n* = 18], AFD‐AMPH [acute food deprivation without AMPH, *n* = 15], and treatment: AL + AMPH [ad libitum fed with AMPH, *n* = 12], FS + AMPH [food‐sated with AMPH, *n* = 13], CFD + AMPH [chronic food‐deprivation with AMPH, *n* = 12], AFD + AMPH [acute food‐deprivation with AMPH, *n* = 15]). For motor neuron activity analysis, only treatment groups were used (before and after treatment analysis), with groups AL + AMPH (*n* = 9), FS + AMPH (*n* = 7), CFD + AMPH (*n* = 7), and AFD (*n* = 6). All experiments were performed on different occasions with time of the day for the experiments was uniform across. For all four feeding states and treatments (−AMPH, +AMPH), different larval clutches were used and were never reused for other conditions.

**FIGURE 1:**
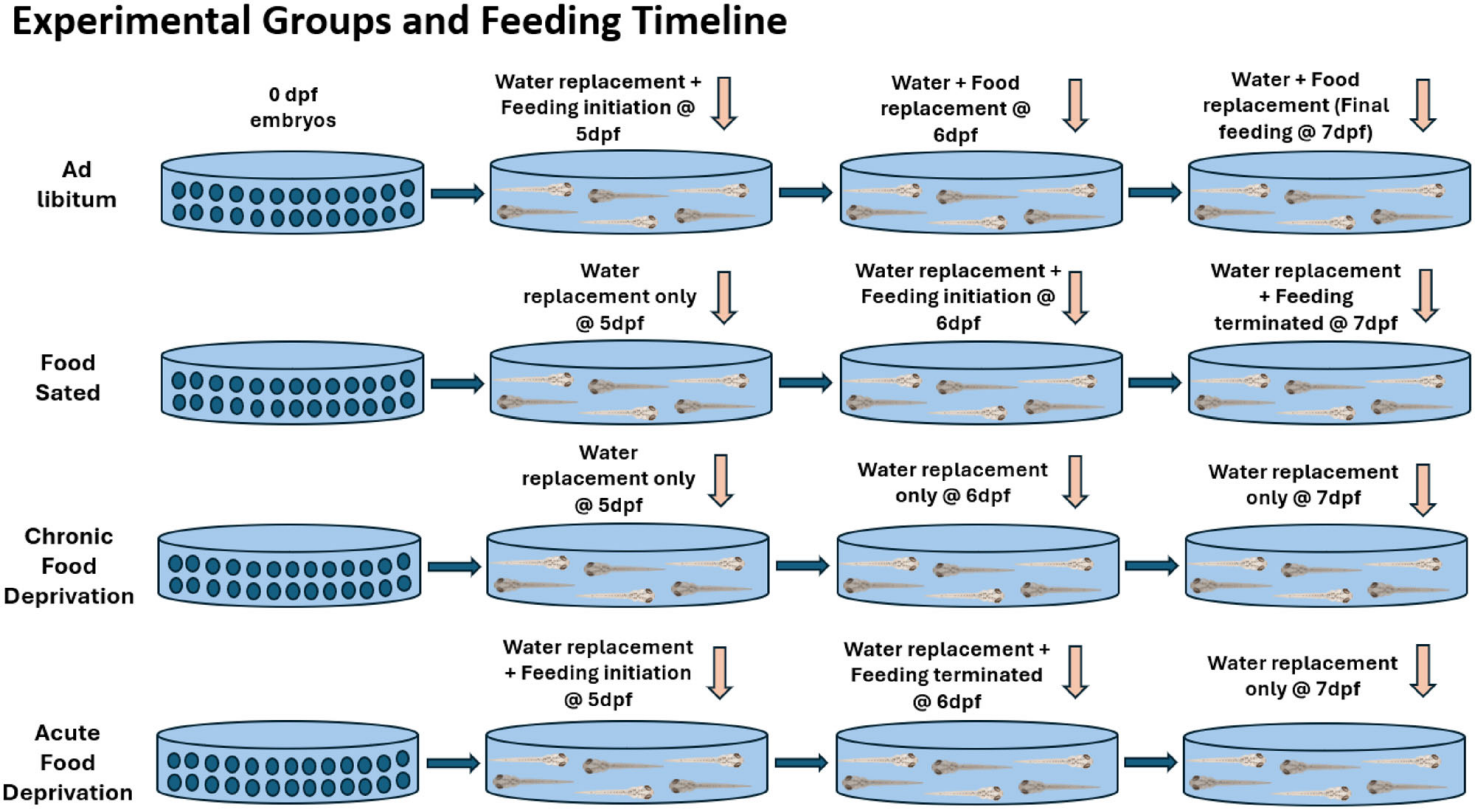
Experimental groups and feeding timeline. We used four different caloric states, namely, ad libitum (AL), chronic food deprivation (CFD), acute food deprivation (AFD), and food‐sated (FS). All larvae used here were 7 dpf at the time of the experiment. (a) In the ad libitum fed (AL) state, food was available to AL larvae when they turned 5 dpf and remained available until the experiments commenced (7 dpf). (b) In the FS state, larvae were deprived of food initially, and food was only made available to them when larvae turned 6 dpf, which remained available till the start of the experiment. Food was available to them only 18 h (till 7 dpf) before the experiment is termed as food‐sated state. (c) In the CFD state, larvae were deprived of food since birth and were never fed before or during the experiment. (d) In the AFD state, feeding started at 5 dpf, where food was removed when larvae turned 6 dpf and were transferred to a dish without food 18 h before the experiments. Here, 18‐h food deprivation is termed “acute food deprivation.”

In the AL state, dry powdered Gemma‐75 fish food (Skretting, USA) was available ad libitum when they turned 5 dpf and remained available until the experiments were started at 7 dpf. FS larvae were deprived of food until they turned 6 dpf, and food was made available until they turned 7 dpf till the start of the experiment. The food was not replaced at 7 dpf of age and was available to them for 18 h in FS state and water was replaced right before the experiments were started. The larvae in the CFD state were deprived of food since they were born and were never fed at any point during the study. AFD larvae were fed till 6 dpf, and larvae were transferred to a dish without food 24 h before the experiments. An 18‐h food deprivation in this study is termed acute food deprivation (Figure 1). Locomotor activity and motor neuron activity were recorded in both groups. These groups were further treated with a dose of 0.7 µM of AMPH (detailed procedure mentioned below). Previous studies performed in zebrafish larvae involving treatment with AMPH demonstrated a dose‐dependent behavioral response that showed a “U” pattern where locomotor activity decreased in low and high doses and increased in the moderate dose (0.7 µM) (Irons et al. 2010). Hence, the findings of this and our previous study were the reason for selecting 0.7 µM as a dose of choice. It would be interesting to see how different caloric states alter this significant motor response.

### 2.2 Locomotor Activity Setup and Post‐Processing

**FIGURE 2:**
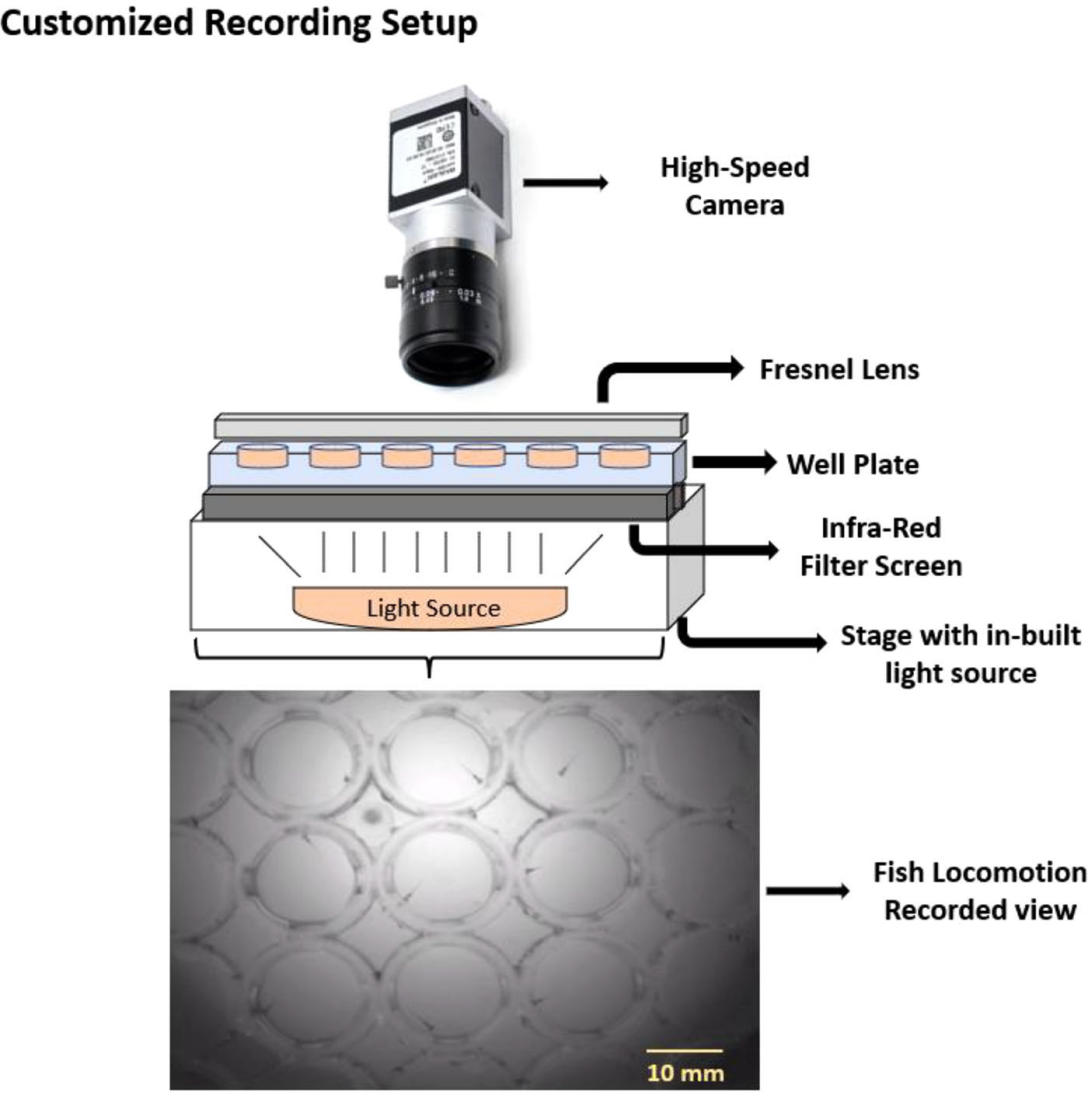
Experimental setup. This customized setup consisted of an invertedly placed high‐speed camera that records the locomotor activity of zebrafish larvae placed in the well plate. An IR filter screen was placed between the plate and the light source to allow IR light to pass and block all other wavelengths. The well plate was placed on top of the in‐built light source of a dissection microscope with an eyepiece, and the upper light source was removed for easy recording. The plate was covered with a Fresnel lens to minimize field distortion.

Wild‐type (abwt) larvae were used for locomotion measurement and analysis. Larvae which did not move past a 50 mm threshold in 10 min or with any visual anomalies were excluded from the study. In Figure 2, we used a dismantled part of the dissection microscope with an in‐built light source to construct a customized setup. An infrared (IR) filter screen (43954; Edmund Optics, NJ, USA) was placed on the light source, allowing only IR frequency light to pass through. Zebrafish larvae exhibited hyper‐locomotion in the dark environment, measured using IR light, and larvae did not adversely affect fish behavior (Basnet et al. 2019). Thus, using IR light to mimic the dark environment would be interesting to examine its increased response in all four states and with AMPH treatment. We placed a 48‐well plate on the IR filter, where larvae were placed individually in each well. A 6.7″ × 6.7″ Fresnel lens (46614; Edmund Optics, NJ, USA) was placed over the 48‐well plate to minimize distortion during recording activities from the wells from the corners. The setup was recorded by an invertedly placed high‐speed camera (Basler Gencam1 GigE camera, 4.5–12 mm, IR ½″). Activity recording was analyzed and generated using Ethovision XT16 (Noldus Information Technology, VA, USA). Generated data were binned within the software, followed by statistical analysis.

Initially, both −AMPH and +AMPH groups were recorded for two 10‐min epochs (baseline, treatment). Before recording, larvae could acclimate to the well environment for 5 min. The baseline epoch in −AMPH groups were recorded for 10 min in fresh fish water. After this recording period, the fish water was pipetted out and replaced by fish water, and activity was recorded for another 10 min (treatment epoch for −AMPH group). Like the −AMPH group, the baseline epoch was similar in time and manner for 10 min. It was followed by pipetting out the fish water and pipetting in 0.5 m: of 0.7 µM solution of D‐amphetamine hemisulfate (Sigma‐Aldrich, MO, USA). A period of 2–3 min was permitted for larvae to consume the drug in the well, followed by another 10‐min recording period (treatment epoch for +AMPH group). This 2–3‐min period was kept uniform in −AMPH group for the sake of uniformity in the recording conditions. Further, each epoch is binned into five 2‐min intervals, and comparisons between binned epochs are made.

### 2.3 Calcium Imaging of Motor Neurons

#### 2.3.1 Sample Preparation

Motor neuron activity was measured in a transgenic zebrafish line Tg(mnx1:GCaMP5) that expressed fluorescence in spinal motor neurons. Larvae were immobilized using 30 µL of 300 µM pancuronium bromide solution (P1918, 10MG; Millipore Sigma, WI, USA), a paralyzing agent to inhibit any voluntary or involuntary activity and contraction for stable neuron recording. This procedure was followed by completely immobilizing the larvae in a 1.5% agarose gel drop. Gel drop is covered with fish water/AMPH according to epochs (baseline, treatment) in −AMPH and +AMPH groups, and recording manner and periods remained the same as in locomotor activity.

#### 2.3.2 Calcium Activity Recording Setup

An inverted epifluorescence microscope was used to record motor neuron activity, consisting of a high‐speed sCMOS camera, a bandpass filter, and a 40× water immersion objective. The microscope setup was connected to an X‐cite 120 fluorescence excitation lamp setup (Excelitas Technologies Corp., Canada). GCaMP6 is a genetically encoded calcium indicator, and it has an excitation/emission wavelength of 488 nm/510 nm; thus, a GFP bandpass filter (Chroma Technology Corp., VT, USA) was installed to obtain the required excitation wavelength. A 40× water immersion objective (Olympus Corporation, Japan) was used through which the excitation light passes and was used to image the fluorescence‐emitting motor neurons (Figure 7a). To record the neuron spiking activity, a Hamamatsu Orca Flash 4.0 high‐speed sCMOS camera (Hamamatsu Photonics, Japan) at 35 fps was used. The recording setup was connected to HCImage Live, a recording and visualization software (Hamamatsu Photonics, Japan), which was used to record and export the recordings in a TIF file format.

#### 2.3.3 Calcium Signal Normalization and Post‐Processing

TIF file frames were opened using ImageJ visualization and analysis software (National Institute of Health). Regions of Interest (ROIs) (single ROI encapsulating all the neurons in the field of view) were selected using a polygon selection tool around the neurons’ soma region, and their respective frame versus intensity data was generated within ImageJ. The data were exported to Microsoft Excel, where frames were converted to time, and this time versus intensity data was further exported into Origin Pro (Version 2023; OriginLab Corporation, Northampton, MA, USA). Data were smoothened using an FFT filter algorithm with window points = 30. From the smoothed data, the baseline was calculated using an in‐built function called “peak analyzer” followed by the “subtract baseline” option with customizable parameters specific to recording quality. The obtained baseline was subtracted from smoothed intensity data and divided by baseline to obtain normalized data. Using the peak analyze option, the find peaks option was used along with a height threshold of 5% to get peak information. The “peak info” option was used to get the peak data (*x*‐axis: time points, *y*: normalized intensity). Inter‐spike intervals (ISIs) were calculated by subtracting the subsequent *x* (time) value from the previous time value correlated to the *y* (peak) data.

### 2.4 Statistical Analysis

Locomotor data were analyzed using GraphPad Prism (version 9.5.1, San Diego, CA, USA). Binned data were analyzed using one‐ and two‐way ANOVA with repeated measures followed by Dunnet's multiple comparisons post‐hoc tests. Total distance and ISI data were analyzed using two‐ and three‐way ANOVA with repeated measures, and Bonferroni and Tukey's post‐hoc tests were used for multiple comparisons. ISI and ROC curves were analyzed using R programming and Python for motor neuron analysis. Raster plots were created using Origin Pro (Version 2023; OriginLab Corporation, Northampton, MA, USA). Statistics were performed with *p < 0.05, **p < 0.01, ***p < 0.001, *p* < 0.0001, and nonsignificant relationships were not shown with any indication. Bars were represented with mean ± SEM.

## 3 Results

### 3.1 Food Deprivation and Satiation Conversely Alter Locomotor Activity Post‐AMPH Treatment

**FIGURE 3:**
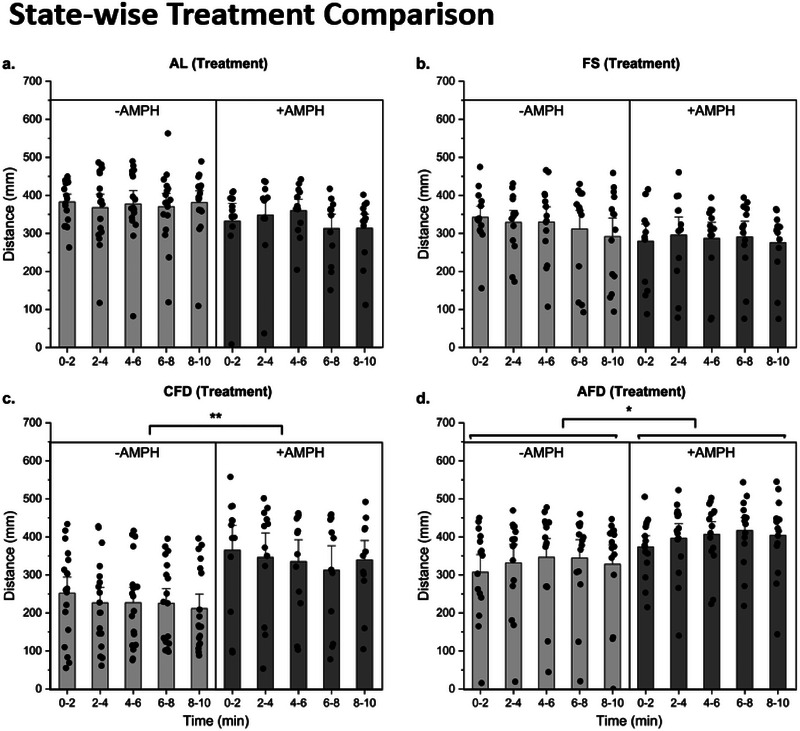
State‐wise treatment comparison. Intervals of treatment epochs were compared in all four feeding states. Two‐way RM ANOVA was used to study treatment, intervals and their interaction. (a) AL larvae did not show a significant change in within intervals and between treatment epochs. (b) Locomotion in FS state also remained unchanged both between epochs and within intervals. **(c)** CFD larvae exhibited a significant change (*p* < 0.01) between −AMPH and +AMPH epochs of treatment groups only. (d) AFD larvae showed a significant increase within intervals in treatment epochs (*p* < 0.05).

For locomotor analysis, we first compared the treatment epochs of −AMPH (no drug added) and +AMPH (with drug) using two‐way RM ANOVA, repeated over intervals in each epoch (Figure 3). In AL state (Figure 3a), we did not find any significant effect imposed by intervals (*F*(4,8) = 1.352, *p* > 0.05), epochs (*F*(1,11) = 1.143, *p* > 0.05) and their interaction (*F* (4,8) = 1.792, *p* > 0.05) locomotion. Post hoc comparison did not show any significant change either. In FS state (Figure 3b), no significant effect of epochs (*F*(1,12) = 0.850, *p* > 0.05), intervals (*F*(4,9) = 1.922, *p* > 0.05), and epoch x interval interaction (*F*(4,9) = 2.509, *p* > 0.05) were observed and post hoc comparison also exhibited a non‐significant change in activity. On the other hand, CFD larvae (Figure 3c) showed a significant effect of treatment epochs (*F*(1,11) = 13.992, *p* < 0.01) and effects of intervals (*F*(4,8) = 0.720, *p* > 0.05); their interaction (*F*(4,8) = 1.459, *p* > 0.05) remained nonsignificant. Here, post hoc comparisons showed epoch significance within all intervals (*p* < 0.05) except for 6–8‐min interval indicating an increase in activity in drug‐treated epoch. Larvae in AFD state (Figure 3d) showed a significant effect of intervals (*F*(4,11) = 3.86, *p* < 0.05) only and no significant effect was shown by epochs (*F*(1,14) = 3.37, *p* > 0.05) and their interaction (*F*(4,11) = 1.352, *p* > 0.05). The multiple comparison test indicated a significant increase within all intervals except in the 4–6‐min interval. The findings suggest that drug's effect was significantly potentiated in food‐deprived states (chronic hunger overall and in acute hunger in a time‐elapsed manner).

**FIGURE 4:**
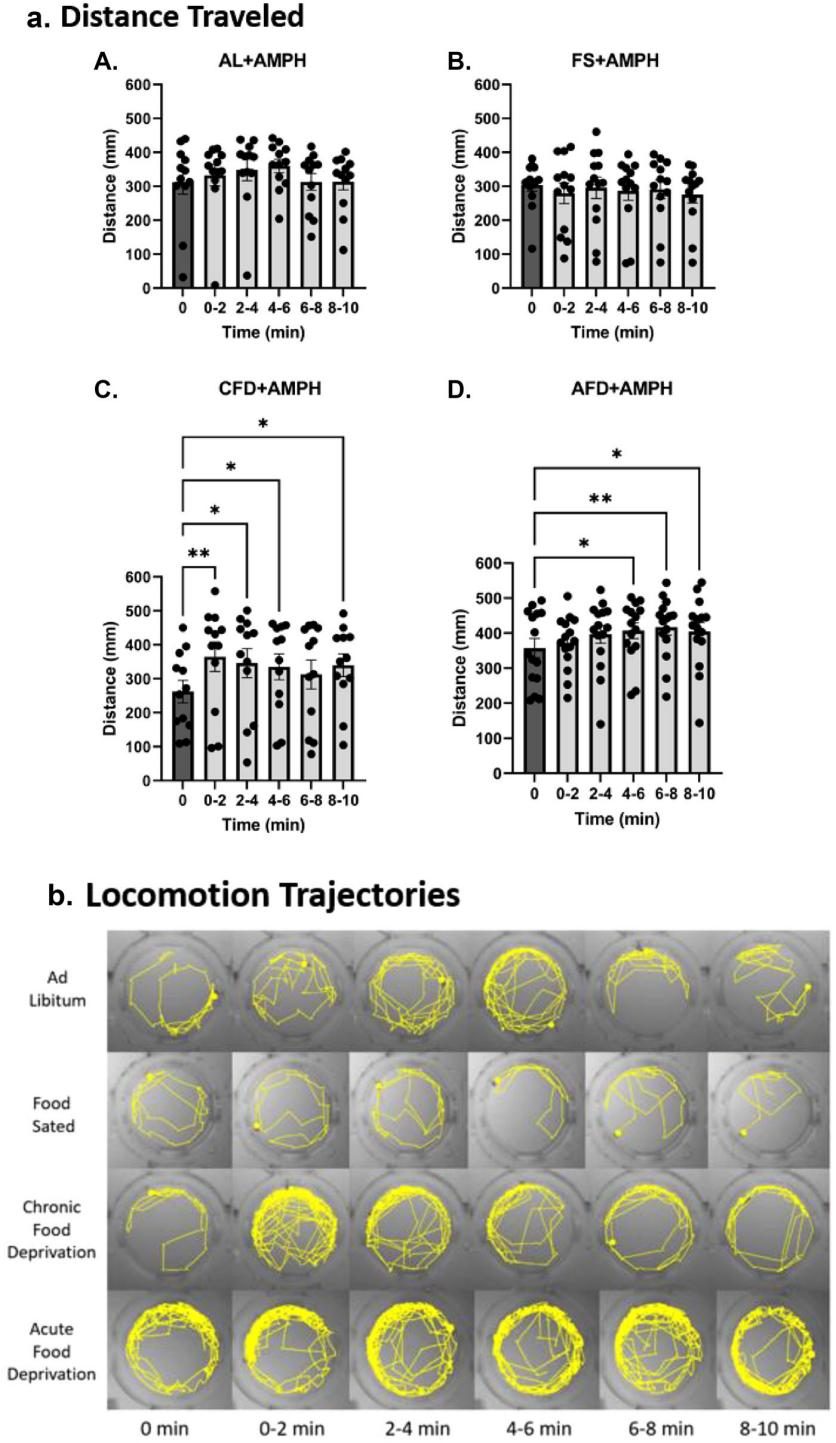
(a) Time‐elapsed locomotion response to amphetamine. The time‐elapsed effect of amphetamine was assessed in binned 2‐min intervals (10 min in total), and all intervals were compared to a 2‐min interval recorded before the addition of amphetamine. The initial 0‐min interval is a 2‐min control interval without amphetamine (dark bar), and the rest (light bars) are amphetamine treated 2‐min intervals compared to the control interval. (A) Distance change observed in the AL state after AMPH was not significant in all five intervals compared to the control (0 min) interval (*p* > 0.05). (B) FS state showed a nonsignificant change in distance traveled by larvae after AMPH treatment compared to a 2‐min control interval (*p* > 0.05). (C) Larvae in the CFD state showed the most significant increase instantaneously after AMPH treatment in 0–2 intervals (*p* < 0.01), and the locomotion remained significantly high in all intervals compared to the control interval (*p* < 0.05). (D) AFD larvae showed a significant increase in locomotion after a 4‐min delay (first 2‐min interval) and kept increasing till the end (*p* < 0.01). (b) Visual representation of time‐elapsed locomotion trajectories from fish swimming shows the path covered by larvae in the wells before and after drug treatment in their respective well/zones in the well plate.

To obtain a deeper insight into how intervals would affect the locomotion post drug treatment, we analyzed the time‐elapsed swimming (locomotion) activity in all four caloric states and compared a 2‐min control interval (taken from last 2‐min interval of locomotor activity in the baseline epoch) with AMPH‐treated intervals (five intervals each of 2 min) (Figure 4a). Locomotion analysis was performed using RM one‐way ANOVA with intervals as a repeated measure. In the AL state, one‐way ANOVA did not reveal a significant interval effect (*F*(5,55) = 1.589, *p* > 0.05) between intervals (Figure 4aA). The post hoc comparison also did not indicate any significance between intervals post‐AMPH administration. Similarly, in the FS state, the statistical test did not show a significant effect of interval on swimming activity after AMPH treatment (*F*(5,60) = 1.589, *p* > 0.05). Planned comparison results also remained nonsignificant between intervals (Figure 4a,B). Contrarily, chronic food deprivation significantly increased swimming activity in subjects after AMPH treatment (*F*(5,55) = 3.279, *p* < 0.05), and planned comparisons between intervals also revealed a statistical significance across all intervals where AMPH showed an immediate effect after treatment (Figure 4a,C). Larvae in AFD state post‐AMPH treatment showed the most significant impact of intervals among all states (*F*(5,70) = 3.734), *p* < 0.01). Post hoc comparisons indicated a significant increase in locomotor activity between intervals compared to the control interval, and the drug induced a significant locomotor activity after a 4‐min delay (Figure 4a,D). Figure 4b represents the visual representation as swimming tracks of the larvae in the four feeding states over time intervals.

**FIGURE 5:**
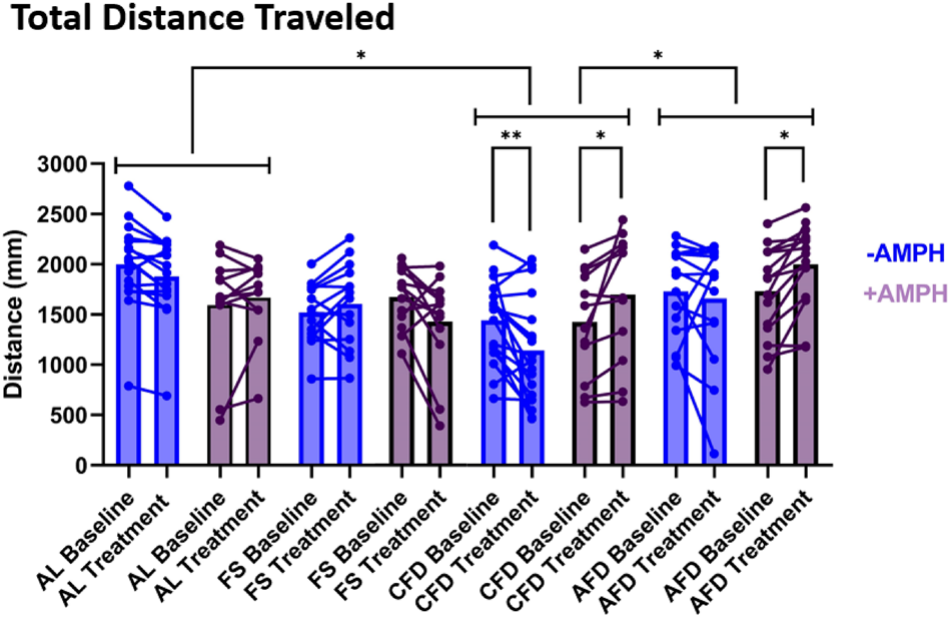
Total distance traveled. Cumulative locomotion was analyzed for two separate 10‐min epochs in both control and AMPH‐treated groups in all four states using three‐way RM ANOVA, and comparisons were made between epochs (state × epoch × drug). Locomotor activity significantly changed between epochs in CFD in the control (*p* < 0.01) as well as the AMPH‐treated group (*p* < 0.05). Cumulatively, control larvae moved significantly shorter distances, whereas AMPH‐treated larvae evoked significant hyperactivity between baseline and treatment epochs. AFD larvae only displayed a significant increase after AMPH treatment only between epochs. AL and FS failed to show significance in control and AMPH treatment conditions. However, control FS larvae moved mildly longer distances, and AMPH treatment caused a moderate non‐significant decrease, indicating that the FS state evokes a hypoactive response to AMPH treatment opposite to CFD and AFD states.

We also measured total locomotor activity over 10‐min epochs (baseline and treatment) for all four states using three‐way RM ANOVA with state, epoch, and drug being independent factors and locomotor activity as a dependent factor (Figure 5). In locomotor activity quantification, we observed significant effects of state (*F*(3,108) = 4.791, *p* < 0.01), epoch × drug interaction (*F*(1,108) = 10.27, *p* < 0.01), and state × drug × epoch interaction (*F*(3,108) = 9.546, *p* < 0.0001). Post hoc comparisons between epochs showed a significant decrease in the −AMPH group of CFD state (*p* < 0.01) and a significant increase in +AMPH groups of CFD and AFD states (*p* < 0.05). After AMPH treatment, the post hoc test showed an increase in locomotor activity in the AFD + AMPH state (*p* = 0.0121) and in the CFD + AMPH state (*p* = 0.0541) between epochs. State‐wise planned comparison revealed an overall significant difference between AL/CFD states and CFD/AFD states (*p* < 0.05) where state‐wise effect between CFD/AFD state was relatively greater (*p* = 0.115) than AL/CFD (*p* = 0.118).

### 3.2 AMPH Treatment Increases Motor Neuron Activity Response and Spiking Frequency in Food‐Deprivation States

**FIGURE 6:**
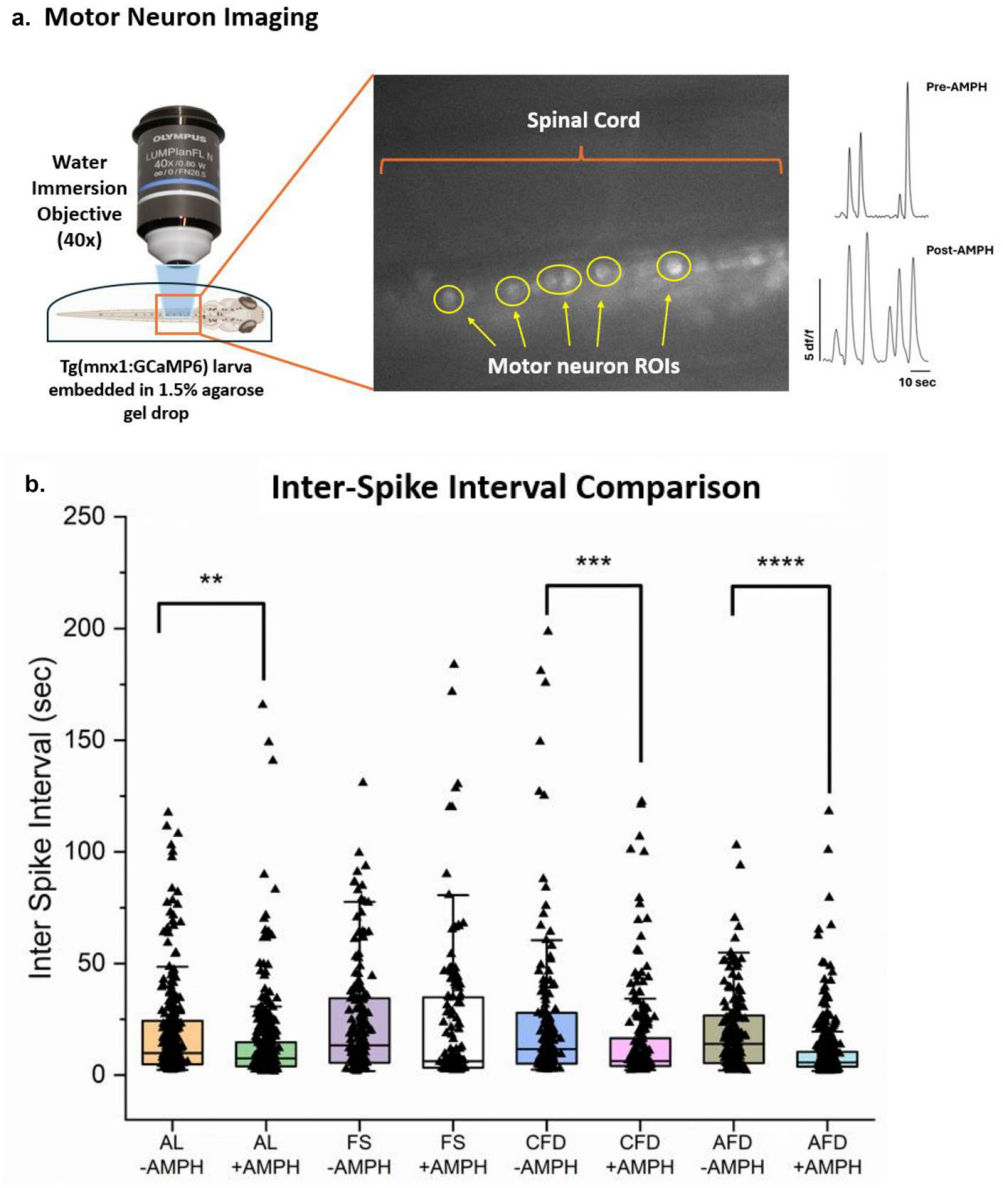
Motor neuron imaging and Raster plot representation for motor neuron spike distribution. (a). A 40× water immersion objective was used to record motor neuron activity in the larval spinal cord while the fish was immobilized and embedded in a drop of 1.5% agarose solution that was covered with fish water. A representation of larval spinal cord and motor neuron ROIs and calcium activity traces are shown. (b) Kruskal–Wallis ANOVA for dependent data showed a significant decrease in inter‐spike interval (an increase in spike occurrence rate) in AL (*p* < 0.01), CFD (*p* < 0.001) and AFD (*p* < 0.0001) state.

Previously, we analyzed the effects of caloric states and AMPH in locomotion (swimming activity). Spinal motor neurons directly affect phenotypic locomotor behavior, influenced by numerous neuron interconnections to and from both identified and yet‐to‐be‐identified. Moreover, physiological states such as food‐deprivation and stimulant drugs such as AMPHs are known to affect specific neuron populations in the brain (Daberkow et al. 2013; Alhadeff et al. 2019; Johansson et al. 2008). Thus, to assess the cohesive effects of AMPH and feeding states, we investigated the spinal motor neuron activity by recording the neuron spiking (Figure 6a) in four different feeding states, followed by acute AMPH treatment. We explored the frequency of motor neuron spiking in terms of ISI activity (Figure 6b). Using Kruskal–Wallis ANOVA with post hoc comparison, we found a significant decrease in ISI of motor neurons in AL state [$\chi_{3}^{2}=92.577$; *Z *= 3.654; median diff: 152.305; *p* < 0.01], CFD state [$\chi_{3}^{2}=92.577$; *Z* = 4.163; median diff: 233.47; *p* < 0.001] and in AFD state [$\chi_{3}^{2}=92.577$; *Z* = 6.131; median diff: 296.108; *p* < 0.0001]. These observations indicate that spiking activity in the AFD state was affected the most by AMPH treatment followed by CFD and AL states whereas the FS state was not affected post‐AMPH administration

**FIGURE 7:**
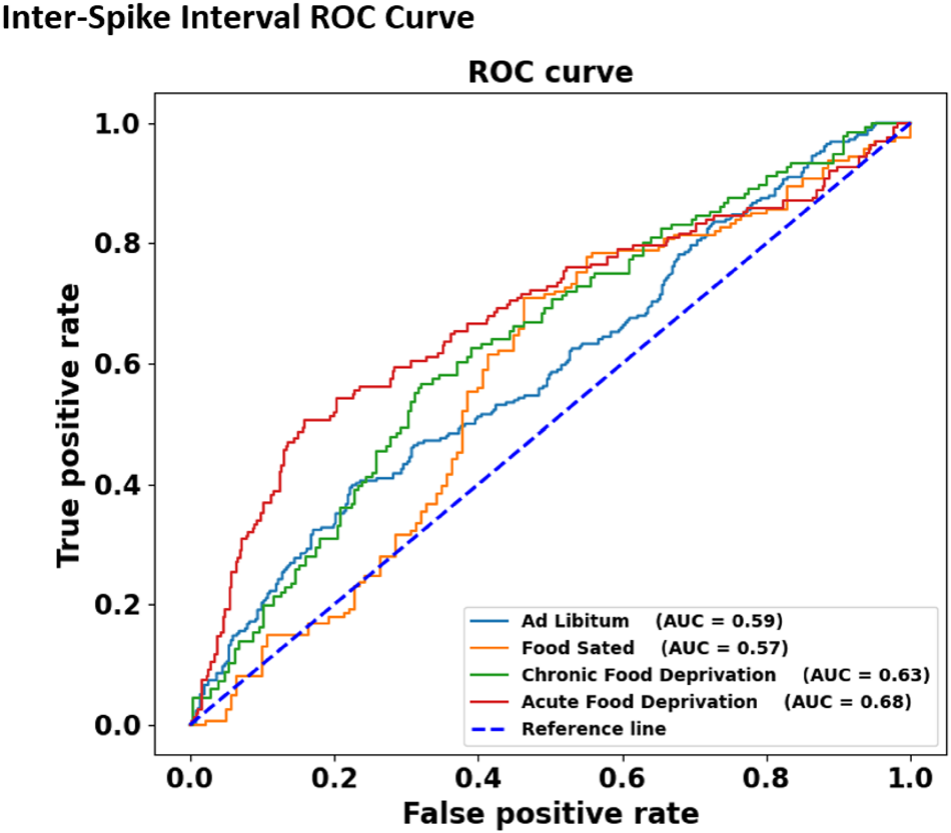
Inter‐spike interval ROC curve. The receiver operator characteristic (ROC) curve demonstrates the difference between neuron activity response in terms of inter‐spike interval in all four states, showing that food‐deprivation states potentiate the effect of amphetamine by increasing neuron spike occurrence rate (lowering inter‐spike frequency) as compared to feeding states.

In Figure 7, a detailed ISI analysis was performed using the ROC curve method that compared the intervals by comparing baseline and treatment epochs to each other and calculated their specific areas under the curve (AUC), representing the latency difference between baseline and treatment epochs in each state. Each AUC peak is calculated by comparing a control and a treatment regime while keeping the control constant. For the AUC to be significant, the AUC peak should rise toward the true positive rate axis relative to the reference line. Overall, the ROC curves provide a statistical overview of the drug treatments indicating the effect of AMPH on ISIs in all four feeding states before and after treatment based on the area under the curve for each state. In the AL state, AMPH treatment showed an area of 0.59 with *p* < 0.001, indicating a significant effect of the drug in decreasing latency of motor neuron spiking. A significant area under the curve of 0.57 was shown by the FS state (*p* < 0.05) in response to the drug; however, the drug in the FS state increased the latency. Chronic food‐deprivation larvae exhibited an area of 0.63 with a significance of *p* < 0.0001) after baseline and treatment comparison. The highest and most significant area under the curve was shown by AFD larvae with an area of 0.68 (*p* < 0.0001). ROC analysis distinguished the ISI between epochs significantly well in both food‐deprivation regimes, highly in the AFD state followed by the CFD state. Area measurement in both feeding states, AL and FS states, was significant; however, the area under the curve was significantly lesser in the AL state, followed by the FS state, compared to food‐deprivation states.

## 4 Discussion

In this study, we quantified the effect of the feeding schedule on AMPH's stimulatory effects by showing the changes in locomotor behavior and spinal motor neuron activity by modeling this scenario in the zebrafish larva. Although previous animal studies provide evidence of hunger–AMPH interactive responses on behavior and the brain, the impact of feeding schedules is yet to be studied. Thus, to explore and understand the meal schedule‐AMPH interaction, we varied the meal schedule of the fish larvae and subjected them to acute AMPH treatment. We hypothesized that locomotion and motor neuron activity would increase in food‐deprivation states and feeding states after AMPH treatment. Our findings suggest that hunger contrastingly modulates AMPH response on motor activity than in feeding states. The outcome of this study could improve our understanding of the interaction between caloric states and AMPH. It would allow zebrafish to be used as a novel animal model to pursue addiction consequence studies further.

Our results show an increase in locomotion by acute and chronic food‐deprived larvae after AMPH treatment relative to fed larvae, which agreed with the other studies (Deroche et al. 1993; De Vaca and Carr 1998; Stuber et al. 2002; Cabeza, Lisa, and Carr 2004; Sharpe, Klaus, and Beckstead 2012). These observations could result from a series of physiological and metabolic changes inside the animal resulting from food stress and drug reward. For example, in vivo animal studies suggest that starvation causes muscles to utilize fatty acids (FAs) as a primary energy source, replacing ketones (Casper 2020; Mehanna, Moledina, and Travis 2008). When FA in the form of supplements in combination with AMPH was fed to rodents, their locomotor activity significantly increased (Trevizol et al. 2013), suggesting FA increased the energy usage and AMPH further potentiated it. In our study, the food we fed the larvae contained oil that served as a source of FA. In addition, fats tend to have a slower metabolism than other macronutrients, resulting in the retention of fats for longer periods (Hargreaves and Spriet 2020; Elia, Stubbs, and Henry 1999). Taking the above studies into account, subjects showing a gradual and lasting increase in locomotion could have a result of slow FA metabolism, increased energy usage, and the well‐known stimulatory effect of AMPH.

The larvae in chronic food deprivation were starved right from their natural dechiornation (a process in which larvae naturally come out of their embryonic eggshell) at 4 dpf. In their chorion, the yolk sac of embryos has a significant supply of lipids and triglycerides converted into FAs (Sant and Timme‐Laragy 2018). After breaking out of their chorions, the FA metabolism could have occurred for a long time due to extended starvation when they were subjected to experimental recordings. Thus, it could be possible that the long‐lasting FA metabolic rate, when interacting with AMPH, abruptly increased the activity for a short time.

The FS state studied here mimics a refeeding regime (where subjects were refed after a short fasting interval). The reason for this assumption sprouts from the fact that larvae in their embryonic stage obtain nutrition from their yolk and depend on human intervention for feeding post‐dechorionation. We assume that FS larvae in this study were initially being fed from their yolk sac. After dechorionation, although the yolk sac was still intact and was providing nutrition to the larva, post‐dechorionation feeding before the recording could have increased the nutritional profile than the yok sac itself mimicking the refeeding. These larvae showed a gradual decrease in locomotion after AMPH treatment. This observation contrasted our hypothesis since food and AMPH are sources of energy and expenditure (Jones et al. 1992). This can be explained from the context of the FA metabolic cascade. A clinical study showed that refeeding rapidly declined FA levels (Kolaczynski et al. 1996). In addition, when rodents with deficient FA were treated with AMPH, they exhibited a decrease in locomotion over time (McNamara et al. 2008). Thus, FA levels may have declined in FS fish in this “refeeding” state, and locomotor activity may have decreased after AMPH treatment. Future studies involving the FA–AMPH interaction in different caloric states could shed light on the cause of this outcome by studying the behavioral responses and metabolism in zebrafish larvae and other animal models.

This study also analyzes the change in motor neuron activity occurring in the spinal cord of zebrafish larvae arising from AMPH treatment in different feeding states. Spinal motor neurons receive projections from multiple regions in the brain. AgRP/NPY neurons, a food intake regulating neuron population essential for energy homeostasis, is an example that gets activated during starvation. NPY neurons also relay inhibitory responses to the spinal cord (Duan et al. 2014; Koch, Acton, and Goulding 2018). Activity in these NPY neurons decreased upon treatment with AMPH (Kobeissy et al. 2008). Furthermore, in mammals, NPY neurons are present with AGRP neurons as a combined AgRP/NPY population, and hunger‐mediated activation in this population induces food‐seeking and locomotion (Mesaros et al. 2008; Essner et al. 2017). In food‐deprived fish, even after the NPY activity increased, AMPH may have shown an overpowering effect over hunger by decreasing the NPY activity post‐AMPH treatment and diminishing the inhibitory effect of NPY toward the spinal cord. Besides this, hypothalamic dopamine neurons also project into the spinal cord, and their excitability is increased during hunger and with AMPH treatment (Puopolo 2019; Barrios et al. 2020; Branch et al. 2013). This could be another explanation for the increased motor neuron activity in the zebrafish spinal cord and needs to be explored further to obtain a concrete explanation.

The spinal cord receives excitatory inputs from the periventricular hypothalamus (PVH) neurons (Ferguson, Latchford, and Samson 2008; Geerling et al. 2010; Sutton et al. 2014). These PVH neurons get excitatory projections from glutamatergic neurons located in the dorsomedial hypothalamus (DMH) when studied in rodents subjected to the fasting‐refeeding state (Imoto et al. 2021). Furthermore, upon treatment with AMPH, glutamatergic neuron transmission was suppressed in rodents (Jones et al. 1992). Therefore, this suppression of glutamatergic neurons in DMH post‐AMPH treatment may have led to the inhibition of neurons present in PVH, further decreasing the transmission into the spinal cord in the fish. Although research does show the connection between glutamatergic neurons and locomotion in aquatic animals (Benvenutti et al. 2021) and suggests that PVH may contribute to locomotor functions in zebrafish (Barrios et al. 2020), future studies in this context would be able to provide insights along with the effects of drugs on these populations in the teleost.

## 5 Conclusion

In conclusion, we investigated the change in stimulatory effects of acutely administered AMPH in zebrafish larvae subjected to four different feeding states. We first measured the change in swimming behavior (distance traveled while swimming) in aforestated conditions. Our results show increased swimming distances traveled by chronic and acute food‐deprived larval groups, which were abrupt but short‐lived in chronic food‐deprived states. At the same time, a gradual prolonged increase in locomotor activity was observed in acute food‐deprivation state after AMPH was administered to them. Ad libitum and FS states had a modest effect on locomotion, with a moderate activity increase and a minor decrease in the latter state post‐AMPH treatment. Similarly, the spiking activity of spinal motor neurons was also analyzed in the abovementioned conditions, and we observed a significant decrement in ISIs in acute and chronic food‐deprivation states and a moderate increase in ad libitum state post‐AMPH administration. AMPH treatment in FS larvae showed an unnoticeable increase in inter‐spike latency. Overall, our findings show the significant potentiation effects of food‐deficit states and a moderate attenuation effect of acute feeding (FS state) on AMPH's characteristic stimulatory effects on locomotor behavior and spinal motor neurons.

## Author Contributions

**Pushkar Bansal**: investigation, data curation, visualization, formal analysis, writing–original draft preparation, software. **Mitchell F. Roitman**: writing–reviewing and editing, data curation, formal analysis, validation, software. **Erica E. Jung**: conceptualization, methodology, writing–reviewing and editing, funding acquisition, project administration, validation, resources.

## Conflicts of Interest

The authors declare no conflicts of interest.

## Ethics Statement

The experiments were approved by The Association for Assessment and Accreditation of Laboratory Animal Care (AAALAC).

### Peer Review

The peer review history for this article is available at https://publons.com/publon/10.1002/brb3.70173.

## Data Availability

Data will be made available upon request to the corresponding author.

## References

[brb370173-bib-0001] Alhadeff, A. L. , N. Goldstein , O. Park , M. L. Klima , A. Vargas , and J. N. Betley . 2019. “Natural and Drug Rewards Engage Distinct Pathways That Converge on Coordinated Hypothalamic and Reward Circuits.” Neuron 103, no. 5: 891–908.e6. 31277924 10.1016/j.neuron.2019.05.050 PMC6728176

[brb370173-bib-0002] Bansal, P. , M. F. Roitman , and E. E. Jung . 2023. “Caloric State Modulates Locomotion, Heart Rate and Motor Neuron Responses to Acute Administration of d‐Amphetamine in Zebrafish Larvae.” Physiology & Behavior 264: 114144. 36889488 10.1016/j.physbeh.2023.114144 PMC10070120

[brb370173-bib-0003] Barrios, J. P. , W. C. Wang , R. England , E. Reifenberg , and A. D. Douglass . 2020. “Hypothalamic Dopamine Neurons Control Sensorimotor Behavior by Modulating Brainstem Premotor Nuclei in Zebrafish.” Current Biology 30, no. 23: 4606–4618.e4. 10.1016/j.cub.2020.09.002. 33007241 PMC7726012

[brb370173-bib-0004] Basnet, R. M. , D. Zizioli , S. Taweedet , D. Finazzi , and M. Memo . 2019. “Zebrafish Larvae as a Behavioral Model in Neuropharmacology.” Biomedicines 7, no. 1: 23. 30917585 10.3390/biomedicines7010023 PMC6465999

[brb370173-bib-0005] Benvenutti, R. , M. Gallas‐Lopes , A. Sachett , et al. 2021. “How Do Zebrafish (*Danio rerio*) Respond to MK‐801 and Amphetamine? Relevance for Assessing Schizophrenia‐Related Endophenotypes in Alternative Model Organisms.” Journal of Neuroscience Research 99, no. 11: 2844–2859. 34496062 10.1002/jnr.24948

[brb370173-bib-0006] Berman, S. M. , R. Kuczenski , J. T. Mccracken , and E. D. London . 2009. “Potential Adverse Effects of Amphetamine Treatment on Brain and Behavior: A Review.” Molecular Psychiatry 14, no. 2: 123–142. 18698321 10.1038/mp.2008.90 PMC2670101

[brb370173-bib-0007] Branch, S. Y. , R. B. Goertz , A. L. Sharpe , et al. 2013. “Food Restriction Increases Glutamate Receptor‐Mediated Burst Firing of Dopamine Neurons.” Journal of Neuroscience 33, no. 34: 13861–13872. 23966705 10.1523/JNEUROSCI.5099-12.2013 PMC3755722

[brb370173-bib-0008] Cabeza, S. , D. V. Lisa , and K. D. Carr . 2004. “A Progressive Ratio Schedule of Self‐Stimulation Testing in Rats Reveals Profound Augmentation of d‐Amphetamine Reward by Food Restriction but No Effect of a ‘Sensitizing’ Regimen of d‐amphetamine.” Psychopharmacology 175: 106–113. 14985931 10.1007/s00213-003-1768-4

[brb370173-bib-0009] Carroll, M. E. , C. P. France , and R. A. Meisch . 1979. “Food Deprivation Increases Oral and Intravenous Drug Intake in Rats.” Science 205, no. 4403: 319–321. https://www.jstor.org/stable/1748263. 36665 10.1126/science.36665

[brb370173-bib-0010] Casper, R. C. 2020. “Might Starvation‐Induced Adaptations in Muscle Mass, Muscle Morphology and Muscle Function Contribute to the Increased Urge for Movement and to Spontaneous Physical Activity in Anorexia Nervosa?” Nutrients 12, no. 7: 2060. 32664448 10.3390/nu12072060 PMC7400818

[brb370173-bib-0011] Cousin, M. A. , J. O. Ebbert , A. R. Wiinamaki , et al. 2014. “Larval Zebrafish Model for FDA‐Approved Drug Repositioning for Tobacco Dependence Treatment.” PLoS One 9, no. 3: e90467. 24658307 10.1371/journal.pone.0090467 PMC3962344

[brb370173-bib-0012] Daberkow, D. P. , H. D. Brown , K. D. Bunner , et al. 2013. “Amphetamine Paradoxically Augments Exocytotic Dopamine Release and Phasic Dopamine Signals.” Journal of Neuroscience 33, no. 2: 452–463. 23303926 10.1523/JNEUROSCI.2136-12.2013 PMC3711765

[brb370173-bib-0013] Deroche, V. , P. V. Piazza , P. Casolini , M. Le Moal , and H. Simon . 1993. “Sensitization to the Psychomotor Effects of Amphetamine and Morphine Induced by Food Restriction Depends on Corticosterone Secretion.” Brain Research 611, no. 2: 352–356. 8334527 10.1016/0006-8993(93)90526-s

[brb370173-bib-0014] De Vaca, S. C. , and K. D. Carr . 1998. “Food Restriction Enhances the Central Rewarding Effect of Abused Drugs.” Journal of Neuroscience 18, no. 18: 7502–7510. 9736668 10.1523/JNEUROSCI.18-18-07502.1998 PMC6793247

[brb370173-bib-0015] Duan, B. , L. Cheng , S. Bourane , et al. 2014. “Article Identification of Spinal Circuits Transmitting and Gating Mechanical Pain.” Cell 159, no. 6: 1417–1432. 10.1016/j.cell.2014.11.003. 25467445 PMC4258511

[brb370173-bib-0016] Elia, M. , R. J. Stubbs , and C. J. K. Henry . 1999. “Differences in Fat, Carbohydrate, and Protein Metabolism Between Lean and Obese Subjects Undergoing Total Starvation.” Obesity Research 7, no. 6: 597–604. 10574520 10.1002/j.1550-8528.1999.tb00720.x

[brb370173-bib-0017] Essner, R. A. , A. G. Smith , A. A. Jamnik , A. R. Ryba , Z. D. Trutner , and M. E. Carter . 2017. “AgRP Neurons Can Increase Food Intake During Conditions of Appetite Suppression and Inhibit Anorexigenic Parabrachial Neurons.” Journal of Neuroscience 37: 8678–8687. 28821663 10.1523/JNEUROSCI.0798-17.2017 PMC5588461

[brb370173-bib-0018] Ferguson, A. V. , K. J. Latchford , and W. K. Samson . 2008. “The Paraventricular Nucleus of the Hypothalamus—A Potential Target for Integrative Treatment of Autonomic Dysfunction.” Expert Opinion on Therapeutic Targets 12, no. 6: 717–727. 18479218 10.1517/14728222.12.6.717 PMC2682920

[brb370173-bib-0019] Fitzgerald, K. T. , and A. C. Bronstein . 2013. “Adderall® (Amphetamine‐Dextroamphetamine) Toxicity.” Topics in Companion Animal Medicine 28, no. 1: 2–7. 10.1053/j.tcam.2013.03.002. 23796480

[brb370173-bib-0020] Geerling, J. C. , J. W. Shin , P. C. Chimenti , and A. D. Loewy . 2010. “Paraventricular Hypothalamic Nucleus: Axonal Projections to the Brainstem.” Journal of Comparative Neurology 518, no. 9: 1460–1499. 20187136 10.1002/cne.22283 PMC2868510

[brb370173-bib-0021] Geuzaine, A. , A. Tyhon , T. Grisar , C. Brabant , B. Lakaye , and E. Tirelli . 2014. “Amphetamine Reward in Food Restricted Mice Lacking the Melanin‐Concentrating Hormone Receptor‐1.” Behavioural Brain Research 262: 14–20. 24412349 10.1016/j.bbr.2013.12.052

[brb370173-bib-0022] Hargreaves, M. , and L. L. Spriet . 2020. “Skeletal Muscle Energy Metabolism During Exercise.” Nature Metabolism 2, no. 9: 817–828. 10.1038/s42255-020-0251-4 32747792

[brb370173-bib-0023] Hernandez, R. E. , L. Galitan , J. Cameron , N. Goodwin , and L. Ramakrishnan . 2018. “Delay of Initial Feeding of Zebrafish Larvae Until 8 Days Postfertilization Has no Impact on Survival or Growth Through the Juvenile Stage.” Zebrafish 15, no. 5: 515–518. 30089231 10.1089/zeb.2018.1579 PMC6198760

[brb370173-bib-0024] Honma, S. , N. Kanematsu , and K. I. Honma . 1992. “Entrainment of Methamphetamine‐Induced Locomotor Activity Rhythm to Feeding Cycles in SCN‐Lesioned Rats.” Physiology & Behavior 52, no. 5: 843–850. 1484838 10.1016/0031-9384(92)90360-e

[brb370173-bib-0025] Hyman, S. E. 1996. “Addiction to Cocaine and Amphetamine.” Neuron 16, no. 5: 901–904. 8630246 10.1016/s0896-6273(00)80111-7

[brb370173-bib-0026] Idemudia, S. O. , and D. E. McMillan . 1984. “Effects of d‐Amphetamine on Spontaneous Motor Activity in Pigeons.” Psychopharmacology 84, no. 3: 315–317. 6440176 10.1007/BF00555205

[brb370173-bib-0027] Imoto, D. , I. Yamamoto , H. Matsunaga , et al. 2021. “Refeeding Activates Neurons in the Dorsomedial Hypothalamus to Inhibit Food Intake and Promote Positive Valence.” Molecular Metabolism 54: 101366. 10.1016/j.molmet.2021.101366. 34728342 PMC8609163

[brb370173-bib-0028] Irons, T. D. , R. C. MacPhail , D. L. Hunter , and S. Padilla . 2010. “Acute Neuroactive Drug Exposures Alter Locomotor Activity in Larval Zebrafish.” Neurotoxicology and Teratology 32, no. 1: 84–90. 19465114 10.1016/j.ntt.2009.04.066

[brb370173-bib-0029] Johansson, A. , R. Fredriksson , S. Winnergren , A. L. Hulting , H. B. Schiöth , and J. Lindblom . 2008. “The Relative Impact of Chronic Food Restriction and Acute Food Deprivation on Plasma Hormone Levels and Hypothalamic Neuropeptide Expression.” Peptides 29, no. 9: 1588–1595. 18550224 10.1016/j.peptides.2008.04.018

[brb370173-bib-0030] Jones, J. R. , W. F. Caul , J. O. Hill , W. F. Caul , and J. O. Hill . 1992. “The Effects of Amphetamine on Body Weight and Energy Expenditure.” Physiology & Behavior 51, no. 3: 607–611. 1523237 10.1016/0031-9384(92)90187-7

[brb370173-bib-0031] Kobeissy, F. H. , J. A. Jeung , M. W. Warren , J. E. Geier , and M. S. Gold . 2008. “Changes in Leptin, Ghrelin, Growth Hormone and Neuropeptide‐Y After an Acute Model of MDMA and Methamphetamine Exposure in Rats.” Addiction Biology 13, no. 1: 15–25. 17910739 10.1111/j.1369-1600.2007.00083.x

[brb370173-bib-0032] Koch, S. C. , D. Acton , and M. Goulding . 2018. “Spinal Circuits for Touch, Pain, and Itch.” Annual Review of Physiology 80: 189–217. 10.1146/annurev-physiol-022516-034303 PMC5891508 28961064

[brb370173-bib-0033] Kolaczynski, J. W. , R. V. Considine , J. Ohannesian , et al. 1996. “Responses of Leptin to Short‐Term Fasting and Refeeding in Humans: A Link With Ketogenesis but Not Ketones Themselves.” Diabetes 45, no. 11: 1511–1515. 8866554 10.2337/diab.45.11.1511

[brb370173-bib-0034] Mabry, P. D. , and B. A. Campbell . 1975. “Potentiation of Amphetamine‐Induced Arousal by Food Deprivation: Effect of Hypothalamic Lesions.” Physiology & Behavior 14, no. 1: 85–88. 1153538 10.1016/0031-9384(75)90146-8

[brb370173-bib-0035] McNamara, R. K. , J. Sullivan , N. M. Richtand , et al. 2008. “Omega‐3 Fatty Acid Deficiency Augments Amphetamine‐Induced Behavioral Sensitization in Adult DBA/2J Mice: Relationship With Ventral Striatum Dopamine Concentrations.” Synapse 62, no. 10: 725–735. 18651642 10.1002/syn.20542

[brb370173-bib-0036] Mehanna, H. M. , J. Moledina , and J. Travis . 2008. “Refeeding Syndrome: What It Is, and How to Prevent and Treat It.” BMJ 336, no. 7659: 1495–1498. 18583681 10.1136/bmj.a301 PMC2440847

[brb370173-bib-0037] Mesaros, A. , S. B. Koralov , E. Rother , et al. 2008. “Activation of Stat3 Signaling in AgRP Neurons Promotes Locomotor Activity.” Cell Metabolism 7, no. 3: 236–248. 18316029 10.1016/j.cmet.2008.01.007

[brb370173-bib-0038] Minassian, A. , J. W. Young , Z. A. Cope , B. L. Henry , M. A. Geyer , and W. Perry . 2016. “Amphetamine Increases Activity but Not Exploration in Humans and Mice.” Psychopharmacology 233, no. 2: 225–233. 26449721 10.1007/s00213-015-4098-4 PMC4703551

[brb370173-bib-0039] Muto, A. , M. Ohkura , T. Kotani , S. I. Higashijima , J. Nakai , and K. Kawakami . 2011. “Genetic Visualization With an Improved GCaMP Calcium Indicator Reveals Spatiotemporal Activation of the Spinal Motor Neurons in Zebrafish.” Proceedings of the National Academy of Sciences of the United States of America 108, no. 13: 5425–5430. 21383146 10.1073/pnas.1000887108 PMC3069178

[brb370173-bib-0040] Puopolo, M. 2019. “The Hypothalamic‐Spinal Dopaminergic System: A Target for Pain modulation.” Neural Regeneration Research 14, no. 6: 925–930. 30761995 10.4103/1673-5374.250567 PMC6404492

[brb370173-bib-0041] Sant, K. E. , and A. R. Timme‐Laragy . 2018. “Zebrafish as a Model for Toxicological Perturbation of Yolk and Nutrition in the Early Embryo.” Current Environmental Health Reports 5, no. 1: 125–133. 29417450 10.1007/s40572-018-0183-2 PMC5876134

[brb370173-bib-0042] Segal, D. S. , and A. J. Mandell . 1974. “Long‐Term Administration of d‐Amphetamine: Progressive Augmentation of Motor Activity and Stereotypy.” Pharmacology Biochemistry and Behavior 2, no. 2: 249–255. 4857295 10.1016/0091-3057(74)90060-4

[brb370173-bib-0043] Sharpe, A. L. , J. D. Klaus , and M. J. Beckstead . 2012. “Meal Schedule Influences Food Restriction‐Induced Locomotor Sensitization to Methamphetamine.” Psychopharmacology 219, no. 3: 795–803. 21750897 10.1007/s00213-011-2401-6 PMC4416415

[brb370173-bib-0044] Shiorring, E. 1980. “Psychopathology Induced by “Speed” Drugs—(Amphetamine, Cocaine and Related Compounds).” Aggressive Behavior 6, no. 3: 260.

[brb370173-bib-0045] Stuber, G. D. , S. B. Evans , M. S. Higgins , Y. Pu , and D. P. Figlewicz . 2002. “Food Restriction Modulates Amphetamine‐Conditioned Place Preference and Nucleus Accumbens Dopamine Release in the Rat.” Synapse 46, no. 2: 83–90. 12211086 10.1002/syn.10120

[brb370173-bib-0046] Sutton, A. K. , H. Pei , K. H. Burnett , M. G. Myers Jr. , C. J. Rhodes , and D. P. Olson . 2014. “Control of Food Intake and Energy Expenditure by Nos1 Neurons of the Paraventricular Hypothalamus.” Journal of Neuroscience 34, no. 46: 15306–15318. 25392498 10.1523/JNEUROSCI.0226-14.2014 PMC4228133

[brb370173-bib-0047] Trevizol, F. , K. Roversi , V. T. Dias , et al. 2013. “Influence of Lifelong Dietary Fats on the Brain Fatty Acids and Amphetamine‐Induced Behavioral Responses in Adult Rat.” Progress in Neuro‐Psychopharmacology and Biological Psychiatry 45: 215–222. 10.1016/j.pnpbp.2013.06.007. 23791617

[brb370173-bib-0048] Wee, C. L. , E. Y. Song , R. E. Johnson , et al. 2019. “A Bidirectional Network for Appetite Control in Larval Zebrafish.” eLife 8: e43775. 10.7554/eLife.43775. 31625906 PMC6799978

[brb370173-bib-0049] Yates, J. W. , J. T. A. Meij , J. R. Sullivan , N. M. Richtand , and L. Yu . 2007. “Bimodal Effect of Amphetamine on Motor Behaviors in C57BL/6 Mice.” Neuroscience Letters 427, no. 1: 66–70. 17920769 10.1016/j.neulet.2007.09.011 PMC2117340

